# Electromagnetic navigation bronchoscopy to access lung lesions in 1,000 subjects: first results of the prospective, multicenter NAVIGATE study

**DOI:** 10.1186/s12890-017-0403-9

**Published:** 2017-04-11

**Authors:** Sandeep J. Khandhar, Mark R. Bowling, Javier Flandes, Thomas R. Gildea, Kristin L. Hood, William S. Krimsky, Douglas J. Minnich, Septimiu D. Murgu, Michael Pritchett, Eric M. Toloza, Momen M. Wahidi, Jennifer J. Wolvers, Erik E. Folch, for the NAVIGATE Study Investigators

**Affiliations:** 1 Inova Health System, Fairfax Hospital, Falls Church, VA USA; 2 East Carolina University, Greenville, NC USA; 3 Pulmonary Department, IIS-Fundacion Jimenez Diaz University Hospital, CIBERES, Madrid, Spain; 4 Department of Pulmonary, Allergy, and Critical Care Medicine and Transplant Center, Cleveland Clinic, Cleveland, OH USA; 5 Medtronic, Minneapolis, MN USA; 6 Pulmonary and Critical Care Associates of Baltimore, Baltimore, MD USA; 7 Division of Cardiothoracic Surgery, University of Alabama at Birmingham, Birmingham, AL USA; 8 Interventional Pulmonology Fellowship Program, The University of Chicago Medicine, Chicago, IL USA; 9 Pulmonary Department, Pinehurst Medical Clinic and FirstHealth Moore Regional Hospital, Pinehurst, NC USA; 10 Department of Thoracic Oncology, Moffitt Cancer Center, Tampa, FL USA; 11 Department of Surgery and Department of Oncologic Sciences, University of South Florida Morsani College of Medicine, Tampa, FL USA; 12 Department of Medicine, Duke University Medical Center, Durham, NC USA; 13 Division of Pulmonary and Critical Care Medicine, Massachusetts General Hospital, Harvard Medical School, 55 Fruit Street, Bulfinch 148, Boston, MA 02114 USA; 14 Present Address: Princeton Baptist Medical Center, Birmingham, AL USA

**Keywords:** Image-Guided Biopsy, Lung Cancer, Lung Neoplasms, Neoplasm Staging, Solitary Pulmonary Nodule

## Abstract

**Background:**

Electromagnetic navigation bronchoscopy (ENB) is an image-guided, minimally invasive approach that uses a flexible catheter to access pulmonary lesions.

**Methods:**

NAVIGATE is a prospective, multicenter study of the superDimension™ navigation system. A prespecified 1-month interim analysis of the first 1,000 primary cohort subjects enrolled at 29 sites in the United States and Europe is described. Enrollment and 24-month follow-up are ongoing.

**Results:**

ENB index procedures were conducted for lung lesion biopsy (*n* = 964), fiducial marker placement (*n* = 210), pleural dye marking (*n* = 17), and/or lymph node biopsy (*n* = 334; primarily endobronchial ultrasound-guided). Lesions were in the peripheral/middle lung thirds in 92.7%, 49.7% were <20 mm, and 48.4% had a bronchus sign. Radial EBUS was used in 54.3% (543/1,000 subjects) and general anesthesia in 79.7% (797/1,000). Among the 964 subjects (1,129 lesions) undergoing lung lesion biopsy, navigation was completed and tissue was obtained in 94.4% (910/964). Based on final pathology results, ENB-aided samples were read as malignant in 417/910 (45.8%) subjects and non-malignant in 372/910 (40.9%) subjects. An additional 121/910 (13.3%) were read as inconclusive. One-month follow-up in this interim analysis is not sufficient to calculate the true negative rate or diagnostic yield. Tissue adequacy for genetic testing was 80.0% (56 of 70 lesions sent for testing). The ENB-related pneumothorax rate was 4.9% (49/1,000) overall and 3.2% (32/1,000) CTCAE Grade ≥2 (primary endpoint). The ENB-related Grade ≥2 bronchopulmonary hemorrhage and Grade ≥4 respiratory failure rates were 1.0 and 0.6%, respectively.

**Conclusions:**

One-month results of the first 1,000 subjects enrolled demonstrate low adverse event rates in a generalizable population across diverse practice settings. Continued enrollment and follow-up are required to calculate the true negative rate and delineate the patient, lesion, and procedural factors contributing to diagnostic yield.

**Trial registration:**

ClinicalTrials.gov NCT02410837. Registered 31 March 2015.

**Electronic supplementary material:**

The online version of this article (doi:10.1186/s12890-017-0403-9) contains supplementary material, which is available to authorized users.

## Background

Guidelines for lung nodule evaluation recommend the least invasive approach given each patient’s clinical presentation [1]. Utilization of electromagnetic navigation bronchoscopy (ENB) has increased over the past ten years as a minimally invasive approach to complement traditional bronchoscopy, endobronchial ultrasound (EBUS), and image-guided transthoracic biopsy. Selection of the most appropriate diagnostic modality based on patient comorbidities and lesion location is critical to provide the fastest, safest, and most complete diagnosis possible.

Seventeen published studies of ENB use have been summarized in three recent meta-analyses [2–4]. Pneumothorax is the most common complication, occurring in approximately 3% of patients [2], lower than the pooled 20% rate reported for transthoracic needle biopsy [5]. However, published studies have typically been small, single-center, retrospective, and mostly conducted by expert users. The safety, usage profile, and clinical utility of ENB in a large, prospective, multicenter, generalizable population is unknown. The pragmatic design [6] of NAVIGATE maximizes patient eligibility, usual care settings, flexibility of adherence, and a relevant primary outcome for clinical practice. The detailed prospective collection of data also minimizes retrospective bias and allows future multivariate analyses to provide more meaningful information on the variable utilization of this technology into real-world practice and its impact on measurable outcomes, such as diagnostic yield and risk. Furthermore, a heterogeneous dataset will be instructive for the design of potential comparative studies with respect to operator training, subject inclusion criteria, data to be collected, definitions, and expected complication rates.

The primary objectives of this protocol-specified 1,000-subject, 1-month interim analysis of the NAVIGATE study [7] are to present the preliminary safety, clinical usage patterns, and performance of ENB in a large, unrestricted, generalizable population across diverse practice settings. The interim data will provide an early look at typical patient and lesion characteristics and procedural standard-of-care, generating questions for future NAVIGATE analyses and new clinical studies. Enrollment and continued follow-up are ongoing.

## Methods

NAVIGATE is a prospective, multicenter, global, single-arm, cohort study in subjects undergoing ENB procedures. Enrollment of up to 1,500 subjects is planned at 37 sites in the United States and Europe. Subjects evaluations occur at baseline (within 30 days of the procedure), on the procedure day, and at 1 month, 12 months, and 24 months post-procedure. This manuscript describes the results of a prespecified 1-month interim analysis of the first 1,000 subjects enrolled at 29 sites in the United States and Europe. Enrollment and 12- and 24-month follow-up are ongoing. Brief methods are included below. A full list of study assessments and definitions is included in Additional files 1 and 2. The study design has been published [7].

Inclusion criteria are intentionally broad to ensure external validity. All consecutive, consented adult patients, who are not pregnant or nursing, and who are candidates for an elective ENB procedure based on physician discretion per recommended guidelines and institutional standard-of-care, are eligible. A maximum of 75 subjects is allowed per site. All investigators must have prior ENB experience. Investigators without extensive experience may enroll a maximum of five “roll-in” cases, which are excluded from this interim analysis. Roll-in cases will be included in the 1-year and 2-year analyses of the full enrollment when a more complete evaluation of the impact of user experience on diagnostic yield and other outcomes can be conducted.

All ENB procedures use the superDimension™ navigation system [8, 9] version 6.0 or higher (Medtronic, Minneapolis, MN) per product instructions and institutional standard practice. All complementary tools and procedures, including choice of catheter and biopsy tools, order of biopsy tool use, and strategy for staging and diagnostic bronchoscopy were performed at clinician discretion and were captured prospectively for data analysis.

The primary endpoint is pneumothorax related to the ENB index procedure rated Grade ≥2 according to the validated Common Terminology Criteria for Adverse Events (CTCAE) scale [7, 10], as adjudicated by an independent medical monitor. Pneumothorax was protocol-specified as the primary endpoint because it is applicable to all ENB procedures, including lung lesion biopsy, lymph node biopsy, fiducial placement, and pleural dye marking. Major secondary endpoints were all ENB-related pneumothorax, bronchopulmonary hemorrhage, and respiratory failure. Other secondary endpoints reported at 1 month were subject self-reported satisfaction with the procedure; adequacy of samples for molecular testing and mutation type; accurate fiducial placement as assessed by follow-up imaging; and success rate of pleural dye marking demonstrated by surgical resection [7].

Diagnostic yield of the ENB index procedure will be calculated at the 12- and 24-month follow-up as the proportion of subjects with a definitive diagnosis (final pathology of the ENB-aided sample). One-month follow-up in this interim analysis is not sufficient to calculate the true negative rate or diagnostic yield. All lung nodules evaluated during the ENB index procedure will be followed for confirmation. Sensitivity, specificity, negative predictive value, and positive predictive value will be published beginning with the 12-month follow-up.

No sample size calculations were conducted for this single-arm, observational study. Analyses were performed using SAS® Version 9.4 (SAS Inc., Cary, NC). Data are summarized by descriptive statistics, including frequency distributions and cross-tabulations for discrete variables and mean, standard deviation, median, minimum, and maximum values for continuous variables. At least 10% of the data are verified against source files by the sponsor using risk-based monitoring.

## Results

### Participants

**Fig. 1:**
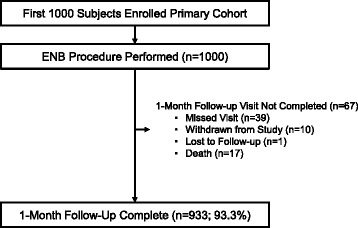
Flow Diagram. As of June 27, 2016, 1,000 primary cohort subjects had been enrolled into the NAVIGATE study and comprise the first protocol-specified interim analysis of the 1-month results. The primary cohort is defined per protocol as those subjects who undergo an ENB index procedure minus roll-in subjects [7]. One-month follow-up is complete in 933/1,000 subjects (93.3%)

This prespecified interim analysis includes the first 1,000 primary cohort subjects enrolled at 29 clinical sites in the United States (27 sites) and Europe (two sites) from April 16, 2015 to June 27, 2016 (Fig. 1, and Additional file 3). Enrollment ranged from 2 to 75 subjects per site. Site types include academic centers (11 sites), private practice (11 sites), and mixed academic/private practice (seven sites). One-month follow-up was completed in 93.3% of subjects. Chronic obstructive pulmonary disease was present in 44.8%. Approximately one-third of subjects had a history of prior invasive lung procedures (Table 1).

**Table 1:** Subject demographics (all primary cohort subjects)

	*N* = 1000 Subjects
Age at consent (years)	67.7 ± 11.3 (1000) [69.0] (21.0–93.0)
Female/Male	49.1%/50.9%
Race	
White	85.5% (855/1000)
Black or African American	12.5% (125/1000)
Asian	0.6% (6/1000)
American Indian or Alaska Native	0.4% (4/1000)
Native Hawaiian or Other Pacific Islander	0.1% (1/1000)
Unknown	0.8% (8/1000)
Unable To Report	0.1% (1/1000)
Hispanic or Latino Ethnicity	4.4% (44/1000)
Tobacco History (Current or Former)	80.8% (807/999)
COPD	44.8% (448/999)
FEV_1_ (% of predicted)	74.8 ± 25.6 (332) [75.5] (20.0–140.0)
FEV_1_/FVC Ratio	0.9 ± 0.2 (331) [0.9] (0.3–1.9)
DLCO (% of predicted)	66.4 ± 24.9 (225) [66.0] (6.0–141.0)
Asthma	12.6% (126/999)
Prior Invasive Lung Procedures^a^	30.6% (306/1000)
Bronchoscopy	20.4% (204/1000)
Standard Bronchoscopy	12.3% (123/1000)
Image-guided Bronchoscopy^b^	9.7% (97/1000)
Transthoracic Needle Aspiration	5.1% (51/1000)
Surgery	11.1% (111/1000)
Other	3.0% (30/1000)
Personal History of Cancer	45.8% (458/999)
Family History of Cancer	61.3% (612/999)
Subject taking Antithrombotic Medications^c^	45.8% (458/1000)
Anticoagulant	10.2% (102/1000)
Prescription Antiplatelet	6.8% (68/1000)
Aspirin	32.5% (325/1000)
Other	1.4% (14/1000)

Data are presented as n/N (%) or mean ± standard deviation (n) [median] (range)

Acronyms: *COPD* chronic obstructive pulmonary disease, *DLCO* diffusing capacity of the lung for carbon monoxide, *FVC* forced vital capacity, *FEV*_*1*_ forced expiratory volume in 1 s, *EBUS* endobronchial ultrasound, *ENB* electromagnetic navigation bronchoscopy

^a^Each subject could have multiple prior procedures

^b^Includes 2.3% (23/1000) standard bronchoscopy with EBUS, 2.8% (28/1000) superDimension ENB, 2.6% (26/1000) superDimension ENB with EBUS, 0.8% (8/1000) other navigation bronchoscopy, and 1.8% (18/1000) other navigation bronchoscopy with EBUS

^c^Subjects could have multiple antithrombotic medications. “Other” includes nonsteroidal anti-inflammatory drugs, fish oil, and vitamins

### Procedural characteristics

**Fig. 2:**
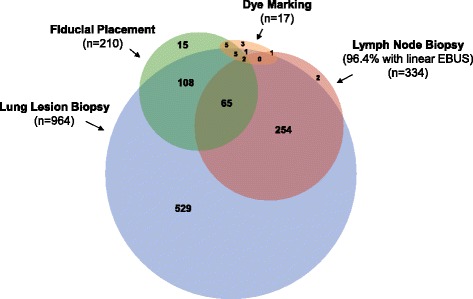
Reasons for Conducting ENB on a Per Subject Basis. The NAVIGATE ENB index procedure could be conducted for more than one purpose in the same anesthetic event, including lung lesion biopsy, fiducial marker placement, pleural dye marking, or lymph node biopsy. Not drawn to scale. Not shown in graph: ENB-guided fiducial marker placement plus lymph node biopsy (*n* = 10); ENB-guided fiducial marker placement plus lymph node biopsy plus ENB-guided pleural dye marking (*n* = 0)

One thousand ENB index procedures were conducted in 1,000 subjects. Procedures were conducted for one or more purposes, including lung lesion biopsy (*n* = 964 subjects), fiducial marker placement (*n* = 210), pleural dye marking (*n* = 17), and/or lymph node biopsy (*n* = 334; primarily guided by linear EBUS), as shown in Fig. 2. General anesthesia was used in 79.7% of subjects. Radial EBUS was used during the ENB index procedure in 54.3% (543/1,000) and fluoroscopy was used in 90.1% (1,017/1,129) of lesions. The median ENB procedure time was 25.0 min (interquartile range 14.0–41.0 min). See Table 2 for other procedural characteristics. Overall, 94.8% (827/872) of subjects reported that their expectations for the procedure we adequately met.

**Table 2:** General procedural characteristics (all primary cohort subjects)

	*N* = 1000 Procedures
General Anesthesia	79.7% (797/1000)
Moderate Sedation	20.3% (203/1000)
ENB Software Version	
Version 6	18.4% (184/1000)
Version 7	81.6% (816/1000)
Radial EBUS used During ENB Procedure^a^	54.3% (543/1000)
Cone Beam CT used	5.4% (54/1000)
Total Procedure Time (Bronchoscope In/Out), min	52.0 (36.0 [35.0–71.0])
ENB Procedure Time (Locatable Guide In/Out), min^b^	25.0 (27.0 [14.0–41.0])

Data are presented as n/N (%) or median (interquartile range [Q1-Q3])

Acronyms: *CT* computed tomography, *EBUS* endobronchial ultrasound, *ENB* electromagnetic navigation bronchoscopy

^a^Other than for lymph node biopsy but including all biopsy, fiducial, and pleural dye marking procedures

^b^Data only available for 499 subjects, because question was added to case report forms after enrollment had begun

### Safety

Pneumothorax CTCAE Grade ≥2 occurred in 32/1,000 subjects (3.2%; Table 3). Any-grade pneumothorax occurred in 49 subjects (4.9%). Bronchopulmonary hemorrhage was 1.0% CTCAE Grade ≥2 (10/1,000) and 2.3% overall (23/1,000). CTCAE Grade ≥4 respiratory failure occurred in 6/1,000 (0.6%). As of the 1-month follow-up (which allowed a visit window up to 37 days post-ENB), 23 subjects had died (six completed the 1-month visit; 17 did not). No deaths were considered related to the ENB device or associated tools by either the clinical investigator or the independent medical monitor. There was one procedure-related death due to Grade 5 hypoxic respiratory failure 9 days after the ENB index procedure, deemed related to complications of general anesthesia, in a patient with multiple comorbidities, including cirrhosis, hepatocellular carcinoma, small cell carcinoma, and ovarian cancer.

**Table 3:** Adverse events related to the ENB index procedure or devices (1 Month Follow-up)^a^

	*N* = 1000 Subjects
Pneumothorax	
CTCAE Grade 2 or Higher	3.2% (32/1000)
All Grades	4.9% (49/1000)
Bronchopulmonary Hemorrhage	
CTCAE Grade 2 and Higher	1.0% (10/1000)
All Grades	2.3% (23/1000)
Respiratory Failure, CTCAE Grade 4 or Higher	0.6% (6/1000)
Death (anesthesia-related respiratory failure 9 days post-ENB)^b^	0.1% (1/1000)

Data are presented as % (n/N subjects)

Acronyms: *CTCAE* Common Terminology Criteria for Adverse Events, *ENB* electromagnetic navigation bronchoscopy

^a^Other than expected observations associated with anesthesia (e.g., common or expected post-procedure pain, transient nausea, transient emesis, post-procedure constipation)

^b^Grade 5 hypoxic respiratory failure 9 days after the ENB index procedure, deemed related to complications of general anesthesia, in a patient with multiple comorbidities, including cirrhosis, hepatocellular carcinoma, small cell carcinoma, and ovarian cancer. As of 1-month follow-up, a total of 23 subjects had died (6 completed 1-month follow-up visit; 17 did not). As of the 1-month follow-up, no other deaths were considered related to the ENB device or associated tools by either the clinical investigator or the independent medical monitor

### Lung lesion biopsies

Among the 964 subjects (1,129 lesions) undergoing lung lesion biopsy, the median lesion size was 20.0 mm (interquartile range: 16.0 mm [Q1: 14.0, Q3: 30.0]), and 49.7% of lesions were <20 mm in diameter (Table 4). Most lesions were in the peripheral (62.6%) or middle (30.1%) lung thirds. An airway to the lesion was visible on pre-procedure CT (bronchus sign) in 48.4%.

**Table 4:** Lung lesion characteristics (subjects undergoing ENB- aided biopsy)

	*N* = 1129 Lesions in 964 Subjects
Pre-test probability of malignancy (physician estimation)	67.1 ± 26.5 (790) [75.0] (0.0–100.0)
Pre-test probability of malignancy (Swenson’s equation)^a^	61.6 ± 29.4 (789) [67.1] (2.9–100.0)
Average Lung Lesion Size, mm	
Mean ± SD (N)	23.6 ± 14.4 (1129)
Median, Range (min-max)	20.0 (3.0–118.0)
Interquartile Range (Q1-Q3)	16.0 (14.0–30.0)
< 20 mm	49.7% (561/1129)
≥ 20 mm	50.3% (568/1129)
Lesion Location	
Right Upper Lobe	31.7% (358/1129)
Right Middle Lobe	8.1% (91/1129)
Right Lower Lobe	19.0% (215/1129)
Left Upper Lobe	25.9% (292/1129)
Left Lower Lobe	15.3% (173/1129)
Lung Zone^b^	
Peripheral third of lung on CT	62.6% (707/1129)
Middle third of lung on CT	30.1% (340/1129)
Proximal third of lung on CT	7.3% (82/1129)
Lesion Visible on Fluoroscopy	60.0% (610/1017)
Ground Glass Lesions (Suzuki Class 1 or 2) [28]	6.3% (71/1123)
Spiculated Lesion Border	60.9% (687/1128)
Bronchus Sign Present on CT	48.4% (546/1129)
Lesion PET Positive (≥2.5 standard uptake value)	80.9% (479/592)

Data are presented as n/N (%) or mean ± standard deviation (n) [median] (range)

Acronyms: *CT* computed tomography, *PET* positron emission tomography, *SD* standard deviation

^a^Pre-test probability of malignancy (calculated): x = −6.8272 + (0.0391 * “age”) + (0.7917 * “tobacco history”) + (1.3388 * “history of extrathoracic cancer”) + (0.1274 * “diameter in mm”) + (1.0407 * “spiculation”) + (0.7838 * “upper lobe”). Not applicable to patients with a diagnosis of cancer that has been made within the previous 5 years or to patients with previous lung cancer [29]

^b^See Folch et al. 2016 for definitions [7]

ENB was able to navigate successfully to allow a tissue biopsy (according to subjective operator assessment) in 910 subjects (94.4%) and 1,036 lesions (91.8%; Table 5). Rapid on-site evaluation (ROSE) for immediate peri-procedural feedback on pathology specimens was conducted in 66.1% (601/909) of subjects. Among 247 lesions diagnosed with primary lung adenocarcinoma or non-small-cell lung cancer not otherwise specified, molecular genetic testing was attempted in 70/247 (28.3%), with adequate tissue in 56/70 (80.0%; Table 5).

**Table 5:** Procedural characteristics in lung lesion biopsy cases

	*N* = 1129 Lesions in 964 Subjects
Navigation Success (per lesion)^a^	91.8% (1036/1129)
Navigation Success (per subject)^a^	94.4% (910/964)
Number of Lesions Biopsied (per subject)	1.2 ± 0.5 (964) [1.0] (1.0–5.0)
Biopsy Tools Used During ENB Index Procedure^b^	
Aspiration Needle	52.2% (503/964)
Biopsy Forceps	81.2% (783/964)
Cytology Brush	47.0% (453/964)
Needle-Tipped Cytology Brush	20.2% (195/964)
Triple Needle-Tipped Cytology Brush	23.4% (226/964)
GenCut™ Core Biopsy Tool	18.9% (182/964)
Bronchoalveolar Lavage or Washing	37.8% (364/964)
Rapid on-site evaluation (ROSE) used^c^	66.1% (601/909)
Molecular/genetic testing attempted^d^	28.3% (70/247)
Molecular/genetic testing successful	80.0% (56/70)
Inadequate Sample	20.0% (14/70)
Molecular/genetic testing not attempted^d^	71.7% (177/247)
Not Necessary	49.2% (87/177)
Not Standard Practice	41.2% (73/177)
Other	9.6% (17/177)

Data are presented as n/N (%) or mean ± standard deviation (n) [median] (range)

^a^Able to navigate successfully and allow a tissue biopsy (according to subjective operator assessment)

^b^More than one tool used per procedure

^c^Per subject, among subjects with an ENB-aided tissue sample obtained. Data missing for 1 subject

^d^Per lesion, among 247 lesions with primary adenocarcinoma (*n* = 233) or primary non-small-cell lung cancer not otherwise specified (*n* = 14). More than one reason could be chosen per lesion

**Fig. 3:**
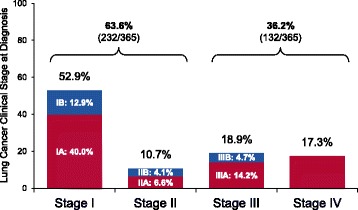
Lung Cancer Clinical Stage at Diagnosis in Subjects with Primary Lung Cancer (365 subjects with 395 lesions). Not shown in graph: One subject had a diagnosis of Stage 0

Based on the final pathology results of the ENB index procedure, tissue was interpreted as malignant in 417/910 (45.8%) subjects (Table 6). Primary lung cancer was diagnosed in 40.1% of subjects. Preliminary clinical stage [11] in subjects diagnosed with primary lung cancer was 52.9% Stage I, 10.7% Stage II, 18.9% Stage III, and 17.3% Stage IV (Fig. 3), to be confirmed with follow-up. Lymph node biopsies were attempted during the ENB index procedure (same anesthetic event) in 33.4% of cases (334/1,000). In 322/334 (96.4%) of these cases, mediastinal staging was conducted using linear EBUS. Lymph node biopsy was guided by ENB in 42 cases (alone or in combination with linear EBUS).

**Table 6:** Pathology result aided by the index ENB procedure^a^

*N* = 910 subjects with navigation complete and tissue sample obtained	
Malignant	45.8% (417/910)
Lung cancer	40.1% (365/910)
Non-Small Cell Lung Cancer (NSCLC)	36.4% (331/910)
Adenocarcinoma	23.5% (214/910)
Squamous Carcinoma	11.4% (104/910)
Other NSCLC	1.5% (14/910)
Small Cell Carcinoma	2.9% (26/910)
Neuroendocrine Carcinoma	1.1% (10/910)
Metastatic Carcinoma of Extrathoracic Origin	4.4% (40/910)
Lymphoma	0.2% (2/910)
Malignant Cells (unable to characterize)	0.9% (8/910)
Other	0.3% (3/910)
Site-Reported Non-Malignant or Inconclusive Results	
Non-Malignant	40.9% (372/910)
Normal Lung Tissue/Bronchial Epithelium	10.4% (95/910)
Benign Non-Specific	21.9% (199/910)
Benign Inflammation	15.3% (139/910)
Other^b^	6.7% (61/910)
Infection	2.9% (26/910)
Bacterial	1.8% (16/910)
Fungal	0.9% (8/910)
Viral	0.2% (2/910)
Granuloma	1.4% (13/910)
Atypical Cells	1.9% (17/910)
Lymphocytes	1.1% (10/910)
Organizing Pneumonia	0.8% (7/910)
Interstitial Lung Disease	0.4% (4/910)
Other^c^	1.0% (9/910)
Inconclusive	13.3% (121/910)

Data are presented as n/N (%) or mean ± standard deviation (n) (range)

Acronym: *ENB* electromagnetic navigation bronchoscopy

^a^Preliminary 1-month results, to be confirmed by subsequent surgery, biopsy, or radiographic follow-up through 2 years as appropriate per clinician’s assessment of the patient’s probability of malignancy. One-month follow-up in this interim analysis is not sufficient to calculate the true negative rate or diagnostic yield

^b^Includes reactive bronchial cells and other nonspecific “benign” diagnoses

^c^Reported as 1 case each of: (1) fibroelastic scar, (2) squamous dysplasia, (3) squamous metaplasia, (4) radiotherapy changes, (5) blood clots post-FNA, (6) asbestos fibrosis, (7) benign metastasizing leiomyoma, (8) focally anthracotic alveolated pulmonary parenchyma, and (9) iron pill aspiration

Based on site-reported assessments of the ENB-aided final pathology sample, tissue was interpreted as non-malignant in 372/910 (40.9%) subjects (Table 6). An additional 121/910 (13.3%) were interpreted as inconclusive. Longer follow-up is required to confirm true versus false negatives and calculate diagnostic yield. At this time, the true prevalence of malignancy in the patient population is unknown.

### Fiducial placement and pleural dye marking

A total of 417 fiducial markers were placed in 210 subjects. Subjective operator assessment of accurate fiducial placement was 208/210 (99.0%) and fiducial markers were still present at follow-up imaging in 192/205 (93.7%). In subjects undergoing fiducial marker placement, ENB-related adverse events included eight pneumothoraces CTCAE Grade ≥2 (3.8%), three respiratory failures CTCAE Grade ≥4 (1.4%), and one bronchopulmonary hemorrhage Grade 1. Pleural dye marking was conducted in 17 subjects, of which 15 (88.2%) were considered adequate for surgical resection.

## Discussion

Lung cancer causes one quarter of all cancer deaths, representing a significant public health problem [12]. While the incidence has declined in concert with decreased smoking prevalence, survival rates have improved little over the past 50–60 years, largely due to a high proportion of late-stage diagnoses with a 5-year survival rate of only 4% [12]. Earlier-stage diagnoses will lead to more meaningful improvements in survival and will require modalities that allow the accurate sampling of smaller, more peripheral lung lesions. The National Lung Screening Trial [13] and screening coverage in select high-risk patients [14] has been projected to increase low-dose CT utilization by over ten million procedures annually [15]. However, an extremely high percentage (96%) of false positive screening results [13, 16] and the risk of unnecessary procedures requires the judicious use of minimally invasive options and a careful balance of the risk-to-benefit ratio for further diagnosis and management [17].

Several current technologies can provide minimally invasive diagnostic evaluation in appropriately selected patients, although each has limitations. PET-CT is often considered the second-line diagnostic option for nodules detected on CT [18], but is typically not reimbursed for screening and does not provide tissue diagnosis. Conventional bronchoscopy is safe but is limited to proximal lesions and has a high non-diagnostic rate, potentially leading to unnecessary invasive procedures in 20–25% of patients, including the use of thoracoscopy for diagnostic wedge resection [19–21]. Image-guided transthoracic biopsy provides high diagnostic accuracy but at the cost of pneumothorax rates averaging 20% (range 4 to 62%) [5] and the need for additional procedures to diagnose and stage mediastinal lymph nodes. ENB provides a minimally invasive platform for peripheral lung lesion sampling, concurrent lymph node staging with linear EBUS, preparation for treatment via fiducial placement or localization via pleural dye marking in a single procedure.

The primary objective of this interim NAVIGATE analysis was to evaluate ENB safety. While published pneumothorax rates are low (3.1% [range 0–13%]) [2], most prior studies are single-center with fewer than 100 subjects [4, 7]. The current analysis demonstrates low pneumothorax, bronchopulmonary hemorrhage, and respiratory failure rates in the context of a large, diverse study cohort and a wide range of user experience levels, confirming the safety of advanced bronchoscopy for the access and sampling of all pulmonary nodules. Pneumothorax was also infrequent following fiducial placement (3.8%), in contrast to rates ranging from 22 to 67% following percutaneous fiducial marking [22], Despite the advanced stage of some of the enrolled subjects, there were only 23 deaths within the 1-month follow-up timeframe, further substantiating the safety of the procedure. Only one death was considered related to the ENB index procedure, due to general anesthesia in a patient with multiple comorbidities, and none were related to the ENB device or associated tools. These results suggest a highly favorable risk-to-benefit ratio for the use of ENB to aid in lung lesion biopsies, particularly given the risk profile of the patients included, with approximately 45% COPD incidence and a relatively high rate of Stage III-IV disease.

A second objective of this analysis was to explore the real-world usage patterns and clinical utility of ENB. The interim results elucidate the rates of general anesthesia use (79.7%), ROSE utilization (66.1%), and concurrent fluoroscopic (90.1%) and radial EBUS (54.3%) guidance. Of note, nearly half of ENB index procedures were conducted for multiple purposes, including 33.4% with lymph node staging (primarily EBUS-guided) and 21.0% with fiducial markers placed. Tissue adequacy for molecular genetic testing was also high (80.0%) and similar to prior studies [23]. These results suggest that, in unrestricted practice settings, ENB is used to diagnose peripheral lung nodules and perform concurrent linear EBUS-guided mediastinal lymph node staging in a single anesthetic event, facilitating a multidisciplinary, comprehensive patient care approach.

A third objective of this interim analysis was to provide a preliminary look at ENB performance. From a patient perspective, all important follow-up cadence and treatment decisions are made within the 30-day window after the diagnostic procedure. At the 1-month time-point, ENB provided a preliminary malignant diagnosis in 45.8% of subjects, including 40.1% with lung cancer. The initial 45.8% malignancy rate in NAVIGATE is consistent with other recent ENB publications reporting malignancy rates of 35–60% [24–27], and is expectedly higher than the 3.7% positive malignancy rate seen in the National Lung Screening Trial [13].

One-month follow-up is not sufficient to calculate the true versus false negative rate or diagnostic yield, as the true prevalence of lung cancer in this population is unknown at this time. All non-malignant pathology findings require confirmation with longer-term follow-up or additional diagnostic procedures, depending upon the pretest probability of malignancy and in accordance with society guidelines [1, 18]. All follow-up procedures and final diagnoses will be captured and reported. Early indicators of clinical stage in NAVIGATE subjects diagnosed with lung cancer also suggest a 64% rate of Stage I-II diagnoses, which are more amenable to surgical intervention for curative intent. In this observational study with consecutive enrollment, approximately 36% of NAVIGATE subjects had Stage III-IV lung cancer. Diagnostic testing of late-stage patients in NAVIGATE may reflect not only a lack of standardization for patient selection but also the changing landscape of personalized medicine and treatment options for Stage III-IV disease. Patient selection for ENB, as well as multivariate predictors of safety and effectiveness, will be explored in future NAVIGATE analyses of the full cohort. This will include an analysis of Stage III-IV cases to explore the patient, lesion, and operator/center factors leading to the inclusion of these cases in the study.

The final objective of this preliminary analysis was to generate questions for future NAVIGATE analyses and comparative studies. Unexpected observations included a high percentage of lesions without a CT bronchus sign (52%) and a relatively low proportion of subjects in whom genetic testing was attempted (28%). While current guidelines recommend genetic testing for only late-stage disease, there is extensive variation between institutions. Tissue requirements for comprehensive molecular testing and the practice of personalized medicine will continue to evolve. Future analyses will describe molecular genetic evaluation in the NAVIGATE cohort in more detail. Other future questions include multivariate predictors of safety and diagnostic yield, factors affecting the need for concurrent radial EBUS or other fluoroscopic guidance, usage patterns of fiducial and pleural dye marking, success rates of various biopsy tools, and cost effectiveness. In this way, NAVIGATE will help to set the benchmark for the ideal ENB patient, and define the procedural techniques contributing to enhanced performance. Whether ENB truly enables a shift to an earlier stage diagnosis, and the impact on long-term patient survival, healthcare utilization, and costs, will also be topics for future NAVIGATE analyses.

### Limitations

This is a nonrandomized, single-arm analysis of 1-month interim results. Longer-term follow-up is required to determine the accuracy of ENB-aided diagnoses, and calculate diagnostic yield. Follow-up through 24 months is in progress. This analysis also evaluates only one navigational bronchoscopy system; other systems are currently available for clinical use.

## Conclusions

This early look at the NAVIGATE results provides information about usage patterns and utility of ENB in a large, unrestricted, generalizable population across diverse practice settings. In the first 1,000 subjects enrolled, 1-month follow-up demonstrates low adverse event rates among a heterogeneous cohort. Continued enrollment and follow-up will demonstrate the negative predictive value and delineate the patient, lesion, and procedural characteristics contributing to diagnostic yield. This preliminary analysis generates questions to be explored in future controlled clinical studies. Further follow-up will also help define objective endpoints to guide future population-based guidelines for intervention.

## Additional files

Additional file 1: Study assessments. (DOCX 52 kb)

Additional file 2: Study definitions. (DOCX 56 kb)

Additional file 3: Study sites enrolling subjects in the 1,000-Patient interim analysis. (DOCX 53 kb)

Additional file 4: Ethics committee approvals. (DOCX 54 kb)

## Abbreviations

CT: Computed tomography
CTCAE: Common Terminology Criteria for Adverse Events
EBUS: Endobronchial ultrasound
ENB: Electromagnetic navigation bronchoscopy
PET: Positron-emission tomography
TTNA: Transthoracic needle aspiration
