# Supplementary material for: Electromagnetic navigation bronchoscopy to access lung lesions in 1,000 subjects: first results of the prospective, multicenter NAVIGATE study
Source: BMC Pulm Med. 2017 Apr 11;17:59. doi: 10.1186/s12890-017-0403-9 (PMC5387322; doi:10.1186/s12890-017-0403-9)
Supplement: Supplementary file 1 — Study assessments. (DOCX 52 kb) [file 12890_2017_403_MOESM1_ESM.docx]

**Additional File 2 – Study Assessments**

Sandeep J. Khandhar MD; Mark R. Bowling MD; Javier Flandes MD; Thomas R. Gildea MD; Kristin L. Hood, PhD; William S. Krimsky MD; Douglas J. Minnich MD; Septimiu D. Murgu MD; Michael Pritchett DO MPH; Eric M. Toloza MD PhD; Momen M. Wahidi MD; Jennifer J. Wolvers BSc; Erik E. Folch MD for the NAVIGATE Study Investigators. Electromagnetic Navigation Bronchoscopy to Access Lung Lesions in 1000 Subjects: First Results of the Prospective, Multicenter NAVIGATE Study

This content has been previously published in its current form under the terms of the Creative Commons Attribution 4.0 International License (<http://creativecommons.org/licenses/by/4.0/>). Source: Folch EE, Bowling MR, Gildea TR, et al. Design of a prospective, multicenter, global, cohort study of electromagnetic navigation bronchoscopy. BMC Pulm Med 2016; 16: 60.

| **Subject Demographics** |
| --- |
| - Age - Sex - Ethnicity - Race |
| **Subject Medical History and Baseline Status** |
| - Prior invasive lung procedures and surgeries - Lung function and diffusing capacity - Antithrombotic medication current and prior status, including duration of any discontinuation - Subject risk factors - Pre-procedure probability of malignancy - Quality of life (EQ-5D) |
| **Lesion Characteristics** |
| - Size - Location - Presence of bronchus sign on computerized tomography (CT) - Lung zone (peripheral, middle, and proximal thirds) - Visibility on fluoroscopy (if applicable) - Positron emission tomography (PET)-positive (yes/no) - Associated lymphadenopathy - Distance to closest fissure - Distance from lesion to pleura - Preprocedure probability of malignancy (investigator assessment) |
| **Procedural Assessments** |
| - Indication for procedure - Anesthesia type - Catheter type - Procedure duration - Imaging used (fluoroscopy, PET, radial endobronchial ultrasound [EBUS]) - Ability to successfully navigate to lesion - Use of associated tools and type (e.g., access tools, biopsy forceps, cytology brush, aspiration needle) - Number of lesions biopsied - Number of lymph nodes biopsied (if applicable) - Placement of fiducial markers (if applicable), type used, indication, and status at follow-up imaging - Surgical resection, including use of dye marker, type used, and adequacy for surgical resection - Diagnosis by both cytologic rapid on-site evaluation (ROSE) and pathology - Cancer type (primary or metastatic), if applicable - Cancer stage, if applicable - Adequacy of sample for molecular testing and mutation type (if applicable) - Lymph node number, station, size, and success in obtaining sample (if applicable) - Number and type of repeat electromagnetic navigational bronchoscopy (ENB) procedures or other biopsies - Other health services (e.g., imaging, transfusion, surgery, emergency room admission, prescriptions) received during admission for index procedure - Hospital admission duration - Adverse events, action taken, relationship to device, and outcome |
| **Follow-up Assessments** |
| - Subject satisfaction (at 1-month follow-up only) - Subject quality of life (EQ-5D) at all follow-up visits - ENB Productivity and Activity Questionnaire (ENB-PAQ) at 1 month visit - All health services (e.g., imaging, transfusion, surgery, emergency room admission, prescriptions) received since last visit - All healthcare services related to lung health since index procedure (e.g., primary care and specialist visits, hospital, emergency room, oncology, radiology, pain management). - All therapeutic and diagnostic procedures and diagnoses related to lung health since last visit - Adverse events, action taken, relationship to device, and outcome |
