# Supplementary material for: Electromagnetic navigation bronchoscopy to access lung lesions in 1,000 subjects: first results of the prospective, multicenter NAVIGATE study
Source: BMC Pulm Med. 2017 Apr 11;17:59. doi: 10.1186/s12890-017-0403-9 (PMC5387322; doi:10.1186/s12890-017-0403-9)
Supplement: Supplementary file 2 — Study definitions. (DOCX 56 kb) [file 12890_2017_403_MOESM2_ESM.docx]

**Additional File 3 – Study Definitions**

Sandeep J. Khandhar MD; Mark R. Bowling MD; Javier Flandes MD; Thomas R. Gildea MD; Kristin L. Hood, PhD; William S. Krimsky MD; Douglas J. Minnich MD; Septimiu D. Murgu MD; Michael Pritchett DO MPH; Eric M. Toloza MD PhD; Momen M. Wahidi MD; Jennifer J. Wolvers BSc; Erik E. Folch MD for the NAVIGATE Study Investigators. Electromagnetic Navigation Bronchoscopy to Access Lung Lesions in 1000 Subjects: First Results of the Prospective, Multicenter NAVIGATE Study

This content has been previously published in its current form under the terms of the Creative Commons Attribution 4.0 International License (<http://creativecommons.org/licenses/by/4.0/>). Source: Folch EE, Bowling MR, Gildea TR, et al. Design of a prospective, multicenter, global, cohort study of electromagnetic navigation bronchoscopy. BMC Pulm Med 2016; 16: 60.

| **Bronchopulmonary Hemorrhage** |
| --- |
| A disorder characterized by bleeding from the bronchial wall and/or lung parenchyma. Degree of severity will be classified according to Common Terminology Criteria for Adverse Events (CTCAE) grade. The incidence of bronchopulmonary hemorrhage related to ENB index procedure rated as Grade 2 or higher according to CTCAE scale will be captured and reported:   - Grade 2: Moderate symptoms; medical intervention indicated - Grade 3: Transfusion, radiologic, endoscopic, or operative intervention indicated (e.g., hemostasis of bleeding site) - Grade 4: Life-threatening respiratory or hemodynamic compromise; intubation or urgent intervention indicated - Grade 5: Death   Bronchopulmonary hemorrhage will also be reported based on a newly validated airway bleeding scale (E. Folch and S. Khandhar, unpublished personal communication; publication in progress). |
| **Diagnostic Yield** |
| Diagnostic yield of the ENB procedure will be calculated on a per-subject basis out of all subjects in whom a diagnostic biopsy is attempted and is defined as the proportion of subjects in whom the ENB procedure yielded a definitive diagnosis. Accuracy of all diagnoses aided by the ENB procedure, as well as nondiagnostic cases, will evaluated based on 2-year clinical follow-up (see below). |
| **Diagnostic Accuracy** |
| Sensitivity, specificity, positive predictive value (PPV), and negative predictive value (NPV) will be calculated as the accuracy of the ENB-aided diagnosis (based on final pathology results) compared to the final 2-year diagnosis based on all available procedures and follow-up.  Where a=true positive, b=false positive, c=false negative, and d=true negative:   - **Sensitivity**: Probability that an ENB-guided biopsy will be positive when malignancy is present (true positive rate): = a / (a+c) - **Specificity**: Probability that an ENB-guided biopsy will be negative when malignancy is not present (true negative rate): = d / (b+d) - **PPV**: Probability that malignancy is present when an ENB-guided biopsy is positive: = a / (a+b) - **NPV**: Probability that malignancy is not present when an ENB-guided biopsy is negative: = d / (c+d) |
| **Lesion Size** |
| Defined as greatest diameter of the target lesion. |
| **Navigation Accuracy** |
| Distance between the tip of the locatable guide and the targeted lung lesion (not done for lymph nodes). |
| **Navigation Success** |
| The proportion of cases in which the operator is able to successfully navigate to the lung target with ENB guidance, based on investigator self-assessment. |
| **Navigation Time** |
| Total time that the locatable guide is used in the subject during the ENB procedure. |
| **Peripheral Lung Lesion** |
| A lesion that is located in the outer third of the lung and difficult to reach by traditional bronchoscopy (see Folch et al. BMC Pulm Med. 2016;16:60). |
| **Pneumothorax** |
| A disorder characterized by abnormal presence of air in the pleural cavity resulting in the collapse of the lung. The primary endpoint is the incidence of pneumothorax related to the ENB index procedure rated as Grade 2 or higher. All grades will be captured as a secondary endpoint. Degree of severity will be classified according to CTCAE grade as follows:   - Grade 1: Asymptomatic; clinical or diagnostic observations only; intervention not indicated. - Grade 2: Symptomatic; intervention indicated (e.g., tube placement without sclerosis). - Grade 3: Sclerosis and/or operative intervention indicated; hospitalization indicated. - Grade 4: Life-threatening consequences; urgent intervention indicated. - Grade 5: Death.   Pneumothorax severity will also be reported based on a newly validated scale that takes into account variance in international standard of care for observation and intervention following asymptomatic pneumothorax and compared to the CTCAE criteria. |
| **Procedure Time** |
| Time between initial introduction of the locatable guide into the body and final removal of the locatable guide. |
| **Repeat Biopsy** |
| Additional biopsy or biopsies conducted after the ENB-guided index procedure, required due to insufficient tissue collection for diagnosis at the index procedure. |
| **Respiratory Failure** |
| A disorder characterized by impaired gas exchange by the respiratory system resulting in hypoxemia and a decrease in oxygenation of the tissues that may be associated with an increase in arterial levels of carbon dioxide. Degree of severity will be classified according to CTCAE grade as follows:   - Grade 4: Life-threatening consequences; urgent intervention, intubation, or ventilatory support indicated. - Grade 5: Death. |
| **Stage** |
| Lung cancer staging will be defined according to the Revised International System for Staging Lung Cancer (TNM) Classification System. |
| **Tissue Adequacy** |
| Tissue adequacy is defined as the proportion of cases in which tissue obtained during the ENB index procedure is adequate for subtyping of lung cancer and molecular testing when appropriate |
