# Supplementary material for: Electromagnetic navigation bronchoscopy to access lung lesions in 1,000 subjects: first results of the prospective, multicenter NAVIGATE study
Source: BMC Pulm Med. 2017 Apr 11;17:59. doi: 10.1186/s12890-017-0403-9 (PMC5387322; doi:10.1186/s12890-017-0403-9)
Supplement: Supplementary file 3 — Study sites enrolling subjects in the 1,000-Patient interim analysis. (DOCX 53 kb) [file 12890_2017_403_MOESM3_ESM.docx]

**Additional File 1 – Study Sites Enrolling Subjects in the 1000-Patient Interim Analysis**

Sandeep J. Khandhar MD; Mark R. Bowling MD; Javier Flandes MD; Thomas R. Gildea MD; Kristin L. Hood, PhD; William S. Krimsky MD; Douglas J. Minnich MD; Septimiu D. Murgu MD; Michael Pritchett DO MPH; Eric M. Toloza MD PhD; Momen M. Wahidi MD; Jennifer J. Wolvers BSc; Erik E. Folch MD for the NAVIGATE Study Investigators. Electromagnetic Navigation Bronchoscopy to Access Lung Lesions in 1000 Subjects: First Results of the Prospective, Multicenter NAVIGATE Study

| **Principal Investigator** | **Site** | **City** | **State or Country** | **Region** |
| --- | --- | --- | --- | --- |
| Arenberg, Douglas | University of Michigan Health Systems | Ann Arbor | MI | USA |
| Bansal, Sandeep | Penn Highlands Healthcare | DuBois | PA | USA |
| Bechara, Rabih | Southeastern Regional Medical Center | Newnan | GA | USA |
| Benzaquen, Sadia | University of Cincinnati Physicians Company, LLC | Cincinnati | OH | USA |
| Bhadra, Krish | Pulmonary Medicine Center of Chattanooga | Chattanooga | TN | USA |
| Bowling, Mark | East Carolina University | Greenville | NC | USA |
| Cicenia, Joe | Cleveland Clinic | Cleveland | OH | USA |
| Ellis, Blesilda | Pulmonary Associates of Mobile, PC | Mobile | AL | USA |
| Flandes, Javier | Hospital Fundación Jiménez Díaz | Madrid | Spain | Europe |
| Gass, G. David | East Texas Medical Center Regional Healthcare System | Tyler | TX | USA |
| Hinze, John David | Seton Medical Center Austin | Austin | TX | USA |
| Krimsky, William | Pulmonary and Critical Care Associates of Baltimore, P.A. | Baltimore | MD | USA |
| Lamprecht, Bernd | AKH Linz | Linz | Austria | Europe |
| LeMense, Greg | Blount Memorial Hospital | Maryville | TN | USA |
| Linden, Philip | University Hospitals Case Medical Center | Cleveland | OH | USA |
| Mahajan, Amit | Inova Fairfax Hospital | Falls Church | VA | USA |
| Mattingley, Jennifer | Gundersen Lutheran Medical Foundation, Inc | La Crosse | WI | USA |
| Minnich, Douglas | University of Alabama at Birmingham | Birmingham | AL | USA |
| Murgu, Septimiu | The University of Chicago | Chicago | IL | USA |
| Murillo, Boris | Providence Health Center | Waco | TX | USA |
| Nason, Katie | UPMC - Shadyside | Pittsburgh | PA | USA |
| Nead, Michael | University of Rochester | Rochester | NY | USA |
| Pritchett, Michael | Pinehurst Medical Clinic, Inc | Pinehurst | NC | USA |
| Rickman, Otis | Vanderbilt University | Nashville | TN | USA |
| Singh, Jaspal | Carolinas HealthCare System | Charlotte | NC | USA |
| Sztejman, Eric | Virtua Medical Group, PA | Marlton | NJ | USA |
| Takubo, Tamejiro | CAMC Health Education and Research Institute, Inc | Charleston | WV | USA |
| Wahidi, Momen | Duke University | Durham | NC | USA |
| Zanchi, Dragos | Pulmonary and Sleep of Tampa Bay | Brandon | FL | USA |
