# Supplementary material for: Electromagnetic navigation bronchoscopy to access lung lesions in 1,000 subjects: first results of the prospective, multicenter NAVIGATE study
Source: BMC Pulm Med. 2017 Apr 11;17:59. doi: 10.1186/s12890-017-0403-9 (PMC5387322; doi:10.1186/s12890-017-0403-9)
Supplement: Supplementary file 4 — Ethics committee approvals. (DOCX 54 kb) [file 12890_2017_403_MOESM4_ESM.docx]

**Additional File 4 – Ethics Committee Approvals**

Sandeep J. Khandhar MD; Mark R. Bowling MD; Javier Flandes MD; Thomas R. Gildea MD; Kristin L. Hood, PhD; William S. Krimsky MD; Douglas J. Minnich MD; Septimiu D. Murgu MD; Michael Pritchett DO MPH; Eric M. Toloza MD PhD; Momen M. Wahidi MD; Jennifer J. Wolvers BSc; Erik E. Folch MD for the NAVIGATE Study Investigators. Electromagnetic Navigation Bronchoscopy to Access Lung Lesions in 1000 Subjects: First Results of the Prospective, Multicenter NAVIGATE Study

| **Ethics Committee / Institutional Review Board Name** | **Site** | **Approval Number** | **Included in 1000-patient analysis** |
| --- | --- | --- | --- |
| Ethikkommission des Landes Oberosterreich | AKH Linz, Linz, Austria | Studie Nr. B-104-15 | Y |
| Comitato Etico Area Vasta Centro | Azienda Ospedaliero Universitaria Careggi, Firenze, Italy | CEAVC OSS 16.248 | N |
| Blount Memorial Hospital IRB | Blount Memorial Hospital, Maryville, TN | IRB# 0017 | Y |
| CAMC Institute Research and Grants Admin | CAMC Health Education and Research Institute, Inc., Charleston, WV | Study Number 15-131 | Y |
| Carolinas HealthCare System | Carolinas HealthCare System, Charlotte, NC | IRB File # 10-15-08E | Y |
| Cleveland Clinic IRB | Cleveland Clinic, Cleveland, OH | CCF IRB# 16-206 | Y |
| Duke Medicine IRB | Duke University, Durham, NC | Pro00063273 | Y |
| University & Medical Center IRB Office | East Carolina University, Greenville, NC | UMCIRB 15-000900 | Y |
| East Texas Medical Center IRB | East Texas Medical Center Regional Healthcare System, Tyler, TX | ETMC Protocol #-687 | Y |
| The Gundersen Clinic, Ltd. Human Subjects Committee/ IRB | Gundersen Lutheran Medical Foundation, Inc., La Crosse, WI | 2-15-11-008 | Y |
| Comite Etico de Investigacion Clinica | Hospital Fundación Jiménez Díaz, Madrid , Spain | None Provided | Y |
| Western Institutional Review Board | Inova Fairfax Hospital, Falls Church, VA | Study Num: 1162420 | Y |
| Comitato Ethico Regionale | IRCCS Azienda Ospedaliera Universitaria San Martino – IST, Genova, Italy | N. Registro 353REG2015 | N |
| Western Institutional Review Board | Ocala Lung and Critical Care, Ocala, FL | Study Num: 1163440 | N |
| El Camino Hospital IRB | Palo Alto Medical Foundation, Mountain View, CA | ECH 15-12 | N |
| Western Institutional Review Board | Penn Highlands Healthcare, DuBois, PA | Study Num: 1157556 | Y |
| Western Institutional Review Board | Pinehurst Medical Clinic, Inc., Pinehurst, NC | Study Num: 1155344 | Y |
| Western Institutional Review Board | Providence Health Center, Waco, TX | Study Num: 1157202 | Y |
| Chesapeake IRB | Pulmonary and Critical Care Associates of Baltimore, P.A., Baltimore, MD | Pro00011869 | Y |
| Patient Advocacy Council | Pulmonary and Sleep of Tampa Bay, Brandon, FL | PAC IRB#: 7-0025-15 | Y |
| Mobile Infirmary IRB | Pulmonary Associates of Mobile, PC, Mobile, AL | IRB Study#: 15.0012 | Y |
| Western Institutional Review Board | Pulmonary Medicine Center of Chattanooga, Chattanooga, TN | Study Num: 1164761 | Y |
| Capital Region Research Ethics Committee | Rigshospitalet – Copenhagen, Kobenhavn, Denmark | H-15017134 | N |
| Land Salzburg Ethikkommission | Salzburger Landesklinik (SALK), Salzburg, Austria | CIP Nummer: COVENBP0475 | N |
| Western Institutional Review Board | Seton Medical Center Austin, Austin, TX | Study Num: 1162569 | Y |
| Western Institutional Review Board | Southeastern Regional Medical Center, Newnan, GA | Study Num: 1157336 | Y |
| Joint Research Management Office | St. Bartholomew's Hospital, London, United Kingdom | IRAS ID: 196286 | N |
| Aura IRB - University of Chicago | The University of Chicago, Chicago, IL | Protocol IRB15-0553 | Y |
| Chesapeake IRB | University Hospitals Case Medical Center, Cleveland, OH | Pro00013521 | Y |
| Ministere De L education Nationale De L Enseignement Superier et de la recherche | University Hospitals of Saint Etienne France, St Etienne, France | CCTIRS N 15.848 | N |
| Western Institutional Review Board | University of Alabama at Birmingham, Birmingham, AL | Study Num: 1159053 | Y |
| Western Institutional Review Board | University of Cincinnati Physicians Company, LLC, Cincinnati, OH | Study Num: 1157291 | Y |
| University of Michigan Medical School IRB | University of Michigan Health Systems, Ann Arbor, MI | Study eResearch ID: HUM00105027 | Y |
| University of Rochester IRB | University of Rochester, Rochester, NY | RSRB: RSRB00057698 | Y |
| Western Institutional Review Board | UPMC - Shadyside, Pittsburgh, PA | Study Num: 1159971 | Y |
| Vanderbilt University IRB | Vanderbilt University, Nashville, TN | IRB# 151145 | Y |
| Virtua General IRB | Virtua Medical Group, PA, Marlton, NJ | None provided | Y |
